# Cancer in pregnancy: an association to make one shiver—highlights from ‘Cancer in pregnancy: 15 years after’, 10–11 October 2019, Milan, Italy

**DOI:** 10.3332/ecancer.2019.981

**Published:** 2019-12-03

**Authors:** Giovanni Codacci-Pisanelli

**Affiliations:** Università degli Studi ‘La Sapienza’ di Roma, 00185 Rome, Italy

**Keywords:** cancer in pregnancy, oncofertility

## Abstract

Although rare, the treatment of pregnant women with cancer remains a challenging situation that requires strict collaboration between different specialities and experts in different fields. Frequent lack of experience and knowledge about this condition could lead to late diagnosis, imprecise management, suboptimal treatment, and foetal and maternal harm. Until recently, the choice for a woman diagnosed with cancer during pregnancy was either to sacrifice the foetus by administering effective treatment to the mother or to risk potential harm to the mother by withholding chemotherapy.

This conference report aims to summarise all different aspects of cancer and pregnancy discussed at this 2-day meeting. Data on the safety (for mother and child) of chemotherapy administered after the first trimester of pregnancy are accumulating together with the recommendation to bring pregnancy as close as possible to its natural duration. Several aspects such as the poor prognosis of breast cancer diagnosed in the year after delivery and the delayed growth of foetuses exposed to chemotherapy despite the quasi-normal duration of pregnancy require further investigation. In this apparently tragic situation, results are excellent and comforting data accumulate so that we can transmit an optimistic feeling to women facing cancer during pregnancy.

In the past, the diagnosis of cancer during pregnancy was one of the greatest tragedies in a woman‘s life. In many instances, the choice was either to sacrifice the foetus by administering effective treatment to the mother or to risk potential harm to the mother by withholding chemotherapy.

Many advances have been made in the last 15 years and this situation, albeit still difficult to manage, has now completely changed: in most instances, the mother’s cancer can be treated during the pregnancy without harming the foetus.

The most common cancers in pregnancy are those with a peak incidence during the woman‘s reproductive period and include cancer of the breast and cervix, melanomas, lymphomas and leukaemias.

As the trend for delaying pregnancy into the later reproductive years continues, this rare association is likely to become more common.

Several medical, psychological, social and ethical issues should be considered when treating a pregnant patient with cancer and a multidisciplinary team of trained professionals should be available within a hub and spoke model to offer each pregnant mother with cancer, to her physician and all members of the oncology team, adequate care and support.

In this 2-day congress, all the different aspects of cancer and pregnancy, including epidemiology, clinical presentation, diagnostic workup and treatment modalities were discussed by a panel of international experts.

If there are two words that medical oncologists would like to keep forever apart they are ‘pregnancy’ and ‘cancer’. Everyone wants to protect a pregnant woman and her baby from any disease, from any negative experience: but ‘the emperor of all maladies’ should definitely stay away from pregnant women. Talking about ‘cancer in pregnancy’ raises a sensation of anguish similar to what we feel while looking at ‘the scream’ by Munch. However, there are different interpretations of this picture, and even if the image is the same, the use of different shades of colour somehow softens the feeling. So we must be grateful to Fedro Peccatori and to Giovanna Scarfone for organising this meeting, which was held at Milan University from 10th October to 11th October 2019 where the audience was exposed to unexpected silver linings related to this association.

All the aspects of cancer during pregnancy were extensively discussed: from epidemiology to the administration of chemotherapy to pregnant women, to psychological support. Researchers with different expertise contributed to the often lively debate: gynaecologists, obstetricians, paediatricians (and specifically neonatologists), psychologists, medical oncologists, etc.

The diagnosis of cancer in a pregnant woman is an uncommon event (approximately one in 1,000 pregnancies) but several speakers indicated that the incidence is increasing due to the higher age at pregnancy. The frequency of tumour types diagnosed during pregnancy reflects the incidence in this young population: breast, cervix, melanoma, haematological malignancies, etc. Speakers, however, focused on those tumours that require systemic treatment (namely localised breast cancer) since it is now widely acknowledged that any kind of surgery (i.e., local treatment) may be administered to pregnant women with no damage to the foetus in any phase of pregnancy.

Systemic chemotherapy consisting of ‘traditional’ anti-proliferative anticancer drugs can be safely administered to the mother with no damage to the foetus but only after the first trimester. As Dr Jennifer Litton (from San Antonio, Texas) reported, when she was pregnant she received a list of substance she should avoid that ranged from caffeine to hot-dogs to alcohol: it really sounds strange to affirm today that a pregnant woman, after the first trimester, can safely receive chemotherapy! This has been demonstrated not only by studying women but also their babies who (as reported by Prof Frederic Amant from Amsterdam, the Netherlands) have been followed up for 20 years.

Several practical aspects of treatment have been discussed by Dr Litton: from the now accepted concept that a pregnant woman should be treated with the same doses and schedules designed for non-pregnant women, to the better tolerance of side effects (such as nausea and vomiting) perhaps due to their psychological situation. It has been reported that the main risk consists of under-treatment caused by the tendency to lower chemotherapy doses and by more subtle pharmacological aspects that require further evaluation. The role of the placenta as a ‘bodyguard’ of the foetus and its increased efficacy during pregnancy have also been addressed but this is a very complex aspect of the investigation. The pivotal role of the placenta has also been invoked when discussing the phenomenon of intra-uterine growth retardation that is often observed in foetuses exposed to chemotherapy *in utero* even when the duration of pregnancy is quasi-normal.

Concerning treatment with target agents (antibodies) during the early phases of pregnancy, which mostly occurs accidentally when the woman is not yet aware of her pregnancy, Prof Hatem Azim from Monterey, Mexico, raised the important point that since both physiological data and the few case reports indicate that in the first weeks of pregnancy there is no passage of antibodies into the foetal circulation and no effect on the baby, it should be possible to reassure mothers accidentally exposed to these drugs in the very first phases and to allow the pregnancy to continue while of course stopping these agents. This should also remind all doctors caring for women with cancer to recommend suitable contraception when indicated.

The mantra of chemotherapy during pregnancy is that the foetus should be allowed to develop in physiological conditions as long as possible. There is no oncological need to induce labour, while obstetrical reasons must be discussed on a case-by-case basis. A shorter duration of pregnancy is actually the main cause of immediate and delayed difficulties in the neonate’s physical and psychological development.

Concerning tumour staging, it is possible to obtain accurate evaluation of distant spread by using diffusion-weighted NMR that does imply the use of radiations and does not require contrast agents.

As already mentioned, a diagnosis of cancer is always devastating, but even more so when this happens during pregnancy! Psychological support to the woman, and to her partner, is therefore of enormous importance and this should possibly extend beyond delivery and also benefit older children if present.

Two main aspects of post-partum assistance have been discussed. In the first 12 months after delivery, there is an increased incidence of breast cancer: this remains an elusive and counterintuitive phenomenon which might be related to the physical involution of the breasts that happens during the weaning phase when the breasts return to their normal status. The second point is that breastfeeding is possible only if the woman does not need any systemic treatment: anticancer drugs and hormonal treatment (and their metabolites) or targeted agents do pass into the milk and may cause toxicity to the baby.

The diagnosis of cancer during pregnancy is luckily an uncommon event, but it is very important that we learn to face it in the best possible way in the interest of the mother and of the baby. It is therefore of the utmost importance that all cases are reported as completely as possible to national or international registers as implemented by the TIGRE study or by the INCIP (www.cancerinpregnancy.org) site.

The main conclusions of the congress are that cancer in pregnancy can often be cured with excellent results, and, in most instances, it will not be necessary to make a choice between the mother’s health and the child’s survival. The woman should receive the best possible treatment, as close as possible (in doses and schedule) to what is given to non-pregnant women, special psychological support is of course required. Pregnancy should continue as long as possible but special attention must be given to the newborn even when delivery occurs at a quasi-normal time and breastfeeding, despite its unquestionable importance, should in most cases be avoided. The child’s outcome seems not to be affected by *in utero* treatment, and this is of course most reassuring information. Since the availability of more data will strengthen our knowledge, it is important to include every possible case in the international registry organised by INCIP.

## Conclusion

Cancer in pregnancy—perhaps the picture is now less sombre. It all started with women accepting the risk of receiving chemotherapy during pregnancy because they wanted to give their babies the best of chances of being born safely while still having their mother with them. As physicians, we are moved to be involved in such an exciting experience, and we should always do our best while listening closely to our patients.

## Conflicts of interest

The author has no conflicts of interest to report.

## Funding statement

There was no funding for this article. The “Cancer in pregnancy: 15 years after” congress had no sponsorship from any company that might have a commercial interest in this field but was supported by Fondazione Internazionale Menarini.

